# Analysis of Basic Features in Dynamic Network Models

**DOI:** 10.3390/e20090681

**Published:** 2018-09-07

**Authors:** Pedro J. Zufiria, Iker Barriales-Valbuena

**Affiliations:** 1 Depto. Matemática Aplicada a las TIC, ETSI Telecomunicación, Universidad Politécnica de Madrid, Avda. Complutense 30, E-28040 Madrid, Spain; 2 Information Processing and Telecommunications Center (IPTC), Universidad Politécnica de Madrid, E-28040 Madrid, Spain

**Keywords:** complex networks, stochastic modelling, entropy, estimation

## Abstract

Time evolving Random Network Models are presented as a mathematical framework for modelling and analyzing the evolution of complex networks. This framework allows the analysis over time of several network characterizing features such as link density, clustering coefficient, degree distribution, as well as entropy-based complexity measures, providing new insight on the evolution of random networks. First, some simple dynamic network models, based only on edge density, are analyzed to serve as a baseline reference for assessing more complex models. Then, a model that depends on network structure with the aim of reflecting some characteristics of real networks is also analyzed. Such model shows a more sophisticated behavior with two different regimes, one of them leading to the generation of high clustering coefficient/link density ratio values when compared with the baseline values, as it happens in many real networks. Simulation examples are discussed to illustrate the behavior of the proposed models.

## 1. Introduction

A large variety of complex systems can be analyzed by constructing a model that relies on some network structure [1,2,3,4]. The model may be dynamical, meaning that the values of some (state) variables do change with time and, depending on the nature of such variables, we can have different types of network models. The first type corresponds to dynamic graphs that follow evolution laws defined explicitly on the network [5,6,7,8]; the second type gathers dynamical systems where the state variables are defined on a network [9,10]; finally, the third type refers to co-evolution models that combine evolving networks and dynamical systems. In the first and third type, the underlying network structure changes with time, defining a time-varying or evolving network [11,12]. In the present work, we first characterize the basic features of some simple models of evolving networks whose evolution does not depend on network structure; the time evolution of these features serves as a reference baseline signature of the behavior of simple models. Then, a model that makes use of network structure is proposed to reflect some real network characteristics. The analysis of this model shows several regimes that indicate a sophisticated behavior; for some regime, the network reaches a high clustering coefficient/link density ratio [13] (when compared to the ratio values of baseline signatures), a common feature in many real networks.

The paper is organized as follows: Section 2 presents the general framework for Dynamic Network Models and their characterization via some basic features, whereas entropy measures are shown in Section 3. Section 4 analyzes some simple evolution models whose basic features’ time evolution serves as a behavior reference baseline. More elaborated evolution models that depend on network structure are studied in Section 5. Simulations of Section 6 comparatively illustrate the time evolution of the different features for the proposed models. Finally, concluding remarks are summarized in Section 7.

## 2. Characterization of Network Sequences via Basic Features

Following [12], discrete-time network evolution over time can be generally defined by a random sequence or trajectory ${\left\{G_{t}\right\}}_{t=0,1,\ldots }$, where each $G_{t}$ can take values *g* from G, being G the set of all possible networks. The analysis of ${\left\{G_{t}\right\}}_{t=0,1,\ldots }$ can be framed by considering it as a stochastic process, whose full characterization may be very complex. In the following, we present some basic features that help for a partial characterization of such stochastic process.

### Time Evolution of Network Features

In some cases, we may be interested in the evolution of some quantifiable properties or features, *f*, of the network, defined as follows (see [14] for details):

(1) $$\begin{array}{cc} f:G & ⟶R^{l}, \end{array}$$

(2) $$\begin{array}{cc} g & ⟶f=f(g), \end{array}$$

where f(g) is the function that computes such quantifiable property (number of links, number of triangles, connectivity, degree of nodes, entropy of degree distribution, etc.) in graph *g*.

Note that, when G is endowed with a probability space, then, under some regularity assumptions on *f*, this function defines a random vector. Therefore, the sequence $f\left(G_{t}\right)\in R^{l}$ defines a vector stochastic process that can be analyzed using standard stochastic process techniques. In the following analysis, we will focus on several of these properties such as the number of links, number of triangles, the connectivity and the degree distribution entropy (a scalar summary of the distribution vector). Since for these cases l=1, the study will boil down to the analysis of scalar stochastic processes. A basic analysis would estimate, for instance, the deterministic sequence of expected values $E[f\left(G_{t}\right)]$.

In the following section, we focus on different entropy measures that can also be employed for characterizing the stochastic process ${\left\{G_{t}\right\}}_{t=0,1,\ldots }$.

## 3. Entropy Measures for Stochastic Processes

The stochastic process ${\left\{G_{t}\right\}}_{t=0,1,\ldots }$ is an indexed sequence of random variables, which can be completely characterized until time instant t=T by its joint probability distribution

(3) $$P(G_{0},G_{1},\ldots ,G_{T})\;.$$

This joint distribution may be quite complex to study and, therefore, we may acquiesce in characterizing part of it. For instance, if we consider $G_{i}$ for a fixed time t=i, this snapshot of the process, also called a cross sectional variable, can be represented by a “static” model such as the ones studied in [14], fully characterized by the marginal distribution of $g_{i}$. Accordingly, when considering entropy measures for characterizing a stochastic process, different distributions associated with such process can be considered, as developed below.

### 3.1. Snapshot Entropy and Entropy of Network Features

The simplest approach focuses on the entropy analysis of cross sectional variables $G_{i}$. Hence, one can define the snapshot entropy of index *i*, $H(G_{i})$, of a stochastic process as the entropy of the *i*-th variable $G_{i}$ of the process

(4) $$H\left(G_{i}\right)=-\sum_{g\in G}p\left(G_{i}=g\right)\log p\left(G_{i}=g\right)\;.$$

When considering a network feature *f*, the entropy of the associated random variable $F_{i}=f\left(G_{i}\right)$ satisfies the condition

(5) $$H\left(G_{i}\right)=H\left(G_{i}|F_{i}\right)+H\left(F_{i}\right)\;,$$

and therefore

(6) $$H\left(F_{i}\right)=H\left(f\left(G_{i}\right)\right)\leq H\left(G_{i}\right)\;,$$

where the equality holds only if *f* is an injection.

Note that $H(G_{i})$ in (4) is not to be confused with the feature mentioned in Section 2.1 called *degree distribution entropy*, associated with a concrete sample of $G_{i}$. For a more detailed explanation of degree distributions in static models, see [14].

The computation of $H(G_{i})$, when performed for every i∈{0,1,…,}, would lead to a deterministic time series ${\left\{H_{t}\right\}}_{t=0,1,\ldots }$ as an alternative partial characterization of the stochastic process ${\left\{G_{t}\right\}}_{t=0,1,\ldots }$.

### 3.2. Trajectory Entropy

Furthermore, one can study the entropy of a whole time period evolution of the process, seen as a sequence of T+1 variables. We define the trajectory entropy ($H_{0}^{T}$) of a T+1-length time period of a stochastic process, as the entropy of the joint probability $P(G_{0},G_{1},\ldots ,G_{T})$:

(7) $$H_{0}^{T}=H\left(G_{0},\ldots ,G_{T}\right)=-\sum_{G^{T+1}}p\left(g_{0},g_{1},\ldots g_{T}\right)\log p\left(g_{0},g_{1},\ldots g_{T}\right)\;.$$

If all $G_{i}$ are independent variables, then:

(8) $$H_{0}^{T}=\sum_{i=0}^{T}H\left(G_{i}\right)\;.$$

Note that, in general, as *T* increases, $H_{0}^{T}$ may increase unbounded.

### 3.3. Normalized Asymptotic Entropy

Finally, one may want to characterize the entropy rate as a normalized entropy measure independent of *T*, which globally characterizes the asymptotic behavior of the stochastic process. This entropy rate is defined as

(9) $$H^{R}=\lim_{T\to \infty }\frac{1}{T+1}H_{0}^{T}\;,$$

whenever such limit exists. Alternatively, we can also compute

(10) $${H^{\prime }}^{R}=\lim_{T\to \infty }H\left(G_{T}\;|\;G_{T-1},G_{T-2},\ldots ,G_{1},G_{0}\right)\;,$$

again when this limit does exist. For strongly stationary processes, both measures (9) and (10) do exist and they are equal.

After presenting these measures, some basic evolution models are illustrated in the next section.

## 4. Basic Evolution Models with a Fixed Number of Nodes: Evolution of Number of Links

Let us consider $G_{V}$ the set of all networks (or graphs) $g_{i}=\left(V,E_{i}\right)$ having a fixed set of nodes $V=\{v_{1},\ldots ,v_{N}\}$, with |V|=N; each $g_{i}\in G$ is then characterized by its corresponding set of links $E_{i}\subset E$ with *E* being determined by *V* as the set of all pairs of nodes (|E|=N2=M).

In this framework, any evolution process ${\left\{G_{t}\right\}}_{t=0,1,\ldots }$ is characterized by the sequence of the corresponding ${\left\{E_{t}\right\}}_{t=0,1,\ldots }$. In addition, since $g_{i}\in G_{V}$ can be represented via its corresponding binary adjacency matrix $A\left(g_{i}\right)\in R^{n}\times R^{n}$, the evolution process can also be characterized as a sequence of adjacency matrices ${\left\{A\left(g_{t}\right)\right\}}_{t=0,1,\ldots }={\left\{A_{t}\right\}}_{t=0,1,\ldots }$.

### 4.1. Evolution of the Number of Links

In general, a complete characterization of ${\left\{G_{t}\right\}}_{t=0,1,\ldots }$ will be very cumbersome. Alternatively, we can partially characterize such process by considering

(11) $$\begin{array}{cc} f:G & ⟶\{0,1,\ldots M\}, \end{array}$$

(12) $$\begin{array}{cc} g_{i} & ⟶f\left(g_{i}\right)\;=\;|E_{i}|\;=\;∥A_{i}{∥}_{1}\;=\;m_{i}\;, \end{array}$$

where *f* is the function that computes the number of links in the network. We can partition the set $G_{V}$ into equivalence classes $C_{k},\;k=0,\ldots M$ so that each class $C_{k}$ gathers all graphs containing *k* links: $C_{k}=\left\{g_{i}\in G:f\left(g_{i}\right)=k\right\}$. Then, we can define a stochastic process ${\left\{M_{t}\right\}}_{t=0,1\ldots }$ with each $M_{t}\in \left\{0,1,\ldots ,M\right\}$ which characterizes the transition between classes, and whose state space represents such equivalence classes (hence, we identify $C_{k}$ with state *k*).

In general, for a given instant of time *i*, based on (5), we have that the snapshot entropy of $G_{i}$ and the entropy of $M_{i}$ will satisfy

(13) $$\begin{array}{c} H\left(G_{i}\right)=H\left(G_{i}|M_{i}\right)+H\left(M_{i}\right) \end{array}$$

and this relationship will help to characterize $G_{i}$ via the analysis of $M_{i}$. Therefore, the following proposed models will be partially characterized by analyzing the associated stochastic process, $M_{t}\in \left\{0,1,\ldots ,M\right\}$, for the evolution of the number of links.

### 4.2. A Simple Structure Independent Evolution Model

We define a simple network evolution process that may serve as a reference baseline for comparison purposes. Given $g_{t}$ (equivalently, $E_{t}$ or $A_{t}$), the next time step network $g_{t+1}$ is generated by randomly selecting a pair of nodes $(v_{i},v_{j})\in E$ so that, if there exists a link between them (i.e., $\left(v_{i},v_{j}\right)\in E_{t}$), such link is removed ($E_{t+1}=E_{t}\backslash \left\{\left(v_{i},v_{j}\right)\right\}$) and, if there is no link between the nodes (i.e., $\left(v_{i},v_{j}\right)\notin E_{t}$), then it is created ($E_{t+1}=E_{t}\cup \left\{\left(v_{i},v_{j}\right)\right\}$). Note that if we consider the adjacency matrix representation $A_{t}$, at each stage of time, an element of the matrix $A_{t}$ is randomly chosen so that its value is changed (from 0 to 1 or vice versa) to derive $A_{t+1}$.

Note that the evolution law is determined by the number of links of $g_{t}$. Therefore, as mentioned above, we will start the analysis of this evolution model by characterizing the time evolution of the number of links. The corresponding $M_{t}\in \left\{0,1,\ldots ,M\right\}$ satisfies:

(14) $$\begin{array}{cc} P(M_{t+1}=1|M_{t}=0) & =1, \end{array}$$

(15) $$\begin{array}{cc} P(M_{t+1}=M-1|M_{t}=M) & =1, \end{array}$$

and for i∈{1,…,M−1}:

(16) $$\begin{array}{cc} P(M_{t+1}=j|M_{t}=i) & =\left\{\begin{array}{cc} 0, & \;\mathrm{if}\;j=i\;\mathrm{or}\;|j-i|>1,\\ \frac{i}{M}, & \;\mathrm{if}\;j=i-1,\\ \frac{M-i}{M}, & \;\mathrm{if}\;j=i+1. \end{array}\right. \end{array}$$

This process is a Markov chain with transition probability matrix

(17) $$\begin{array}{cc} P & =\left[\begin{array}{ccccccc} 0 & \frac{1}{M} & 0 & \cdots & 0 & 0 & 0\\ 1 & 0 & \frac{2}{M} & \cdots & 0 & 0 & 0\\ 0 & \frac{M-1}{M} & 0 & \cdots & 0 & 0 & 0\\ 0 & 0 & \frac{M-2}{M} & \cdots & 0 & 0 & 0\\ \vdots & \vdots & \vdots & \ddots & \vdots & \vdots & \vdots \\ 0 & 0 & 0 & \cdots & \frac{M-2}{M} & 0 & 0\\ 0 & 0 & 0 & \cdots & 0 & \frac{M-1}{M} & 0\\ 0 & 0 & 0 & \cdots & \frac{2}{M} & 0 & 1\\ 0 & 0 & 0 & \cdots & 0 & \frac{1}{M} & 0 \end{array}\right]\;, \end{array}$$

which is known as the Ehrenfest model [15], and which can be similarly interpreted as representing an urn with white and black balls, where we randomly select a ball and change it by another ball with different color, hence representing a sort of discrete-time birth–death Markov process [16] but with finite number of states (two boundary conditions). Many discrete distributions have been obtained by studying urn models and Markov processes [17,18,19]. Note that these models can be seen as a reference baseline since they do not exploit the network structure properties (i.e., the relative location of white balls and black balls).

The left stochastic, tri-diagonal, irreducible matrix *P* of Equation (17) has period 2, but it has a unique eigenvector associated with eigenvalue λ=1. This eigenvector defines the stationary distribution of the process, denoted by $M_{s}$, and it can be easily proved that such distribution is binomial:

(18) P(gs∈Ck)=P(Ms=k)=Mk12M,

so that taking a snapshot of the process for large *t* is equivalent to generating a sample from the Gilbert model [20] with $p=\frac{1}{2}$ or, equivalently, the uniform model with maximum entropy (see [14] for details). Note that, given a number of links $M_{s}=k$, the distribution of $G_{i}|\left(M_{s}=k\right)$ is uniform, each link having probability 1|Ck|=1Mk. Hence, considering (18), the entropy expression provided in (13) becomes

(19) H(Gi)=H(Ms)+H(Gi∣Ms)=−∑kp(k)logp(k)−∑kp(k)log1Mk

(20) =−∑kp(k)logp(k)Mk=M·log2=M=N2,

measuring the entropy in bits.

Concerning the entropy of $M_{t}$, it is known that Ehrenfest model snapshot (relative) entropy at time *t*, defined in terms of the Kullback–Leibler divergence between the distribution and the steady state equilibrium distribution

(21) $$\begin{array}{c} H_{\mathrm{rel}}\left(t\right)=-D_{KL}\left(P\left(t\right)|\right|P_{s})=-\sum_{k=0}^{M}P\left(M_{t}=k\right),\log\frac{P(M_{t}=k)}{P(M_{s}=k)} \end{array}$$

is non-decreasing in time as approaches the maximum value zero, upon the so called *H-Theorem* [21].

### 4.3. Extensions of the Model for Asymmetric Evolution

One can extend the symmetric model provided in (17) with the aim of considering cases in which the network may have an uneven tendency to increase or decrease the number of edges.

Let us consider the following transition behavior from $g_{t}$ to $g_{t+1}$: we start selecting a pair of nodes in network $g_{t}$; if the selected pair already has an associated link, such link is removed with probability $p_{r}$, whereas, if such pair does not have an associated link, a link is added between such pair of nodes with probability $p_{a}$. If no change (removal or addition) happens, the process is repeated until the network undergoes some modification, which is registered in $g_{t+1}$.

Again, if we focus the analysis on the time evolution of the number of links, $M_{t}$, the corresponding transition matrix becomes:

(22) $$\begin{array}{cc} P(p_{r},p_{a}) & =\left[\begin{array}{ccccccc} 0 & \frac{p_{r}}{p_{r}+\left(M-1\right)p_{a}} & 0 & \cdots & 0 & 0 & 0\\ 1 & 0 & \frac{2p_{r}}{2p_{r}+\left(M-2\right)p_{a}} & \cdots & 0 & 0 & 0\\ 0 & \frac{\left(M-1\right)p_{a}}{p_{r}+\left(M-1\right)p_{a}} & 0 & \cdots & 0 & 0 & 0\\ 0 & 0 & \frac{\left(M-2\right)p_{a}}{2p_{r}+\left(M-2\right)p_{a}} & \cdots & 0 & 0 & 0\\ \vdots & \vdots & \vdots & \ddots & \vdots & \vdots & \vdots \\ 0 & 0 & 0 & \cdots & \frac{\left(M-2\right)p_{r}}{\left(M-2\right)p_{r}+2p_{a}} & 0 & 0\\ 0 & 0 & 0 & \cdots & 0 & \frac{\left(M-1\right)p_{r}}{\left(M-1\right)p_{r}+p_{a}} & 0\\ 0 & 0 & 0 & \cdots & \frac{2p_{a}}{\left(M-2\right)p_{r}+2p_{a}} & 0 & 1\\ 0 & 0 & 0 & \cdots & 0 & \frac{p_{a}}{\left(M-1\right)p_{r}+p_{a}} & 0 \end{array}\right]\;. \end{array}$$

The analysis of this system can be simplified if we denote $\frac{p_{r}}{p_{a}}=u$ the *unbalance* coefficient, since the matrix can be reformulated as

(23) $$\begin{array}{cc} P(u) & =\left[\begin{array}{ccccccc} 0 & \frac{u}{u+M-1} & 0 & \cdots & 0 & 0 & 0\\ 1 & 0 & \frac{2u}{2u+M-2} & \cdots & 0 & 0 & 0\\ 0 & \frac{M-1}{u+M-1} & 0 & \cdots & 0 & 0 & 0\\ 0 & 0 & \frac{M-2}{2u+M-2} & \cdots & 0 & 0 & 0\\ \vdots & \vdots & \vdots & \ddots & \vdots & \vdots & \vdots \\ 0 & 0 & 0 & \cdots & \frac{(M-2)u}{(M-2)u+2} & 0 & 0\\ 0 & 0 & 0 & \cdots & 0 & \frac{(M-1)u}{(M-1)u+1} & 0\\ 0 & 0 & 0 & \cdots & \frac{2}{(M-2)u+2} & 0 & 1\\ 0 & 0 & 0 & \cdots & 0 & \frac{1}{(M-1)u+1} & 0 \end{array}\right]\;. \end{array}$$

If u<1, the model has more tendency to add links than to remove them, and vice versa for u>1. The analysis and interpretation of the network behavior can be performed either way due to such symmetry. For instance, if u<1, the model can be interpreted as characterizing the following behavior: if the selected pair in $g_{t}$ has an associated link, this link is removed with probability *u*; if the pair does not have an associated link, then a link is added. Again, the selection procedure is repeated until a link is either removed or added, defining $g_{t+1}$.

It can be proved that the resulting stationary distribution has the form:

(24) Pu(Ms=k)=Mkk·u+M−kM·uk∑i=0MMii·u+M−iM·ui,u∈R+,

which can be seen as a generalization of the binomial distribution $\mathrm{Bin}(\frac{1}{2},M)$ via the new parameter *u*.

Repeating a similar procedure to (19) and (20), the corresponding $G_{i}$ entropy can be computed as

Hu(Gi)=Hu(Ms)+H(Gi∣Ms)=−∑kpu(k)logpu(k)−∑kpu(k)log1Mk=−∑kpu(k)logpu(k)Mk,

which for u=1 becomes Hu=1(Gi)=N2=M.

**Figure 1:**
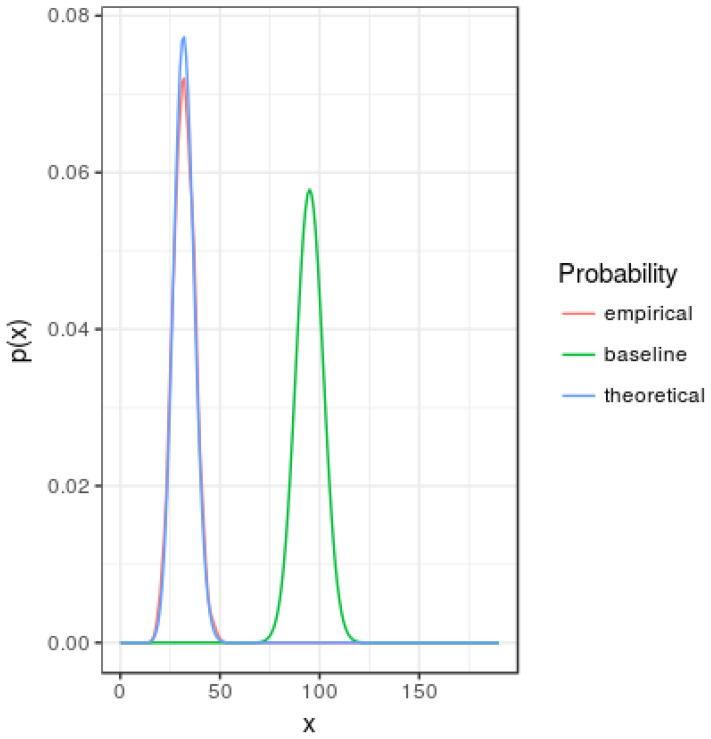
Simple and extended asymmetric models. Comparison among smoothed probability mass functions: baseline, theoretical and empirical for $p_{a}=0.3$ and $p_{r}=1$.

Figure 1 represents smoothed probability mass functions for the baseline, theoretical given by (24) and empirical (based in simulations) with $p_{a}=0.3$ and $p_{r}=1$. Note that asymmetry of the *u* value generates a probability function with less entropy than the corresponding to the baseline mass function.

#### Alternative Simple Model

Another simple model could assume that, whenever an existing edge is selected to be removed, it is removed with probability $p_{r}\in \left[0,1\right]$, whereas, alternatively, a new edge is randomly added. The transition matrix of the corresponding $M_{t}\in \left\{0,1,\ldots ,M\right\}$ for the number of links would be

(25) $$\begin{array}{cc} P & =\left[\begin{array}{ccccccc} 0 & \frac{p_{r}}{M} & 0 & \cdots & 0 & 0 & 0\\ 1 & 0 & \frac{2p_{r}}{M} & \cdots & 0 & 0 & 0\\ 0 & 1-\frac{p_{r}}{M} & 0 & \cdots & 0 & 0 & 0\\ 0 & 0 & 1-\frac{2p_{r}}{M} & \cdots & 0 & 0 & 0\\ \vdots & \vdots & \vdots & \ddots & \vdots & \vdots & \vdots \\ 0 & 0 & 0 & \cdots & \frac{\left(M-2\right)p_{r}}{M} & 0 & 0\\ 0 & 0 & 0 & \cdots & 0 & \frac{\left(M-1\right)p_{r}}{M} & 0\\ 0 & 0 & 0 & \cdots & 1-\frac{\left(M-2\right)p_{r}}{M} & 0 & 1\\ 0 & 0 & 0 & \cdots & 0 & 1-\frac{\left(M-1\right)p_{r}}{M} & 0 \end{array}\right]\;. \end{array}$$

Note that an equivalent symmetric model can be defined as follows. If the selected pair of nodes does not have an associated link, we add such a link with probability $p_{a}$; otherwise, an existing link is removed.

It can be proved that the resulting stationary distribution has the form:

(26) $$\begin{array}{cc} P_{p_{r}}\left(M_{s}=k\right) & =\left\{\begin{array}{cc} \frac{1}{1+\sum_{i=1}^{M}\frac{M\cdot \left(M-p_{r}\right)\cdots \left(M-\left(i-1\right)p_{r}\right)}{i!\;p_{r}^{i}}} & \mathrm{if}\;k=0,\\ \frac{\frac{M\cdot \left(M-p_{r}\right)\cdots \left(M-\left(k-1\right)p_{r}\right)}{k!\;p_{r}^{k}}}{1+\sum_{i=1}^{M}\frac{M\cdot \left(M-p_{r}\right)\cdots \left(M-\left(i-1\right)p_{r}\right)}{i!\;p_{r}^{i}}} & \mathrm{if}\;k\in \{1,\ldots ,M\}, \end{array}\right. \end{array}$$

which can be seen as another generalization of the binomial distribution $\mathrm{Bin}(\frac{1}{2},M)$ via the new parameter $p_{r}\in \left[0,1\right]$. Again, the network snapshot entropy can be computed as

(27) Hpr(Gi)=−∑kppr(k)logppr(k)Mk.

Both models (23) and (25) provide respectively stationary distributions (24) and (26), which, in general, are not binomial. Therefore, if we take a snapshot of these stationary distributions, the resulting network will follow a new static model, different from the standard known reference models for static networks.

Note that again these models can be interpreted as urn-derived finite state discrete-time birth-death models, in the sense that they do not incorporate network structural information, but only the total number of links. In other words, these models do not differentiate among networks that belong to the same equivalence class $C_{k}$, i.e., they are structure independent.

The time evolution of the expected value for the number of links, the clustering coefficient, the connectivity and the sample degree distribution entropy define a vector time series that can be employed as a signature that characterizes the evolution models. The signature of the above considered structure independent models can be employed as a reference baseline to assess more complex behaviors.

In Section 6, these signature quantities are estimated via simulation procedures.

## 5. Evolution Models Depending on Network Structure: Evolution of Clustering Coefficient

Usually, the evolution of networks depends not only on the number of links but also on the network structure. To illustrate this idea, we will analyze the behavior of models whose dynamics depend on the fact that triangles are going to be created or deleted in the network; then, the evolution of the clustering coefficient will be an essential feature to be considered.

Let us consider, for instance, an extension of the asymmetric model of Section 4.3 where the probability of a given link to be added (or removed) may depend on the fact that a triangle will or will not be generated (or eliminated) when adding (or removing) such link. Precisely, the transition behavior from $g_{t}$ to $g_{t+1}$ is defined as follows:

a pair of nodes in network $g_{t}$ is uniformly selected.
  (a) If the selected pair already has an associated link, such link is removed
    i. with probability $p_{r,n}$ when the selected nodes have at least one common neighbor (hence, at least one triangle will be deleted), or
    ii. with probability $p_{r,nn}$ when the selected nodes do not have a common neighbor (no triangle will be deleted);
  (b) if the selected pair does not have an associated link, a link is added between these nodes
    i. with probability $p_{a,n}$ when the selected nodes have at least one common neighbor (hence, some new triangles will be generated), or
    ii. with probability $p_{a,nn}$ when the selected nodes do not have common neighbor (no triangle will be generated).
If no change (removal or addition) happens, the process is repeated until the network undergoes some modification, which is registered in $g_{t+1}$.

The discrete process $g_{t}$ provided by this model remains invariant to a common scaling of all probability values, provided the proportion among them is preserved. Hence, such dynamical model behavior can be reformulated as a function of, for instance, the following three parameters, $\alpha =\frac{p_{a,n}}{p_{a,nn}}$, $\beta =\frac{p_{r,nn}}{p_{r,n}}$ and $u_{1}=\frac{p_{r,n}}{p_{a,n}}$; note that α and β measure the strength for favoring the creation and preservation of triangles, respectively, and the meaning of $u_{1}$ will become clear below. Note that this model is general enough to represent dynamic networks having a tendency to either create (and preserve) or eliminate triangles. In the specific case when α and β are larger than 1, triangle creation (and preservation) are promoted.

The analysis of this model can be complex since the existence and characterization of a stationary behavior may depend on the network size (number of nodes), the selected parameter values and the initial state $g_{0}$. Note that $M_{t}$ cannot be directly defined anymore via a transition matrix of the type of (22), (23) or (25), since the future evolution of such $M_{t}$ depends not only on its actual value but also on some structural properties (i.e., the existence and location of triangles) of $g_{t}$. In addition, the existence and form of a limiting stationary distribution for $M_{t}$ may be a complicated issue to deal with.

### Two Regimes of Behavior

If we assume that $g_{t}$ follows approximately a Gilbert model, the probability $p_{ij,t}$ of any pair of nodes $\left(v_{i},v_{j}\right)$ to have a common neighbor (i.e., they may take part of a triangle) depends on the ratio between the number of links $m_{t}$ and the total number of node pairs M=N2 in $g_{t}$:

(29) $$\begin{array}{c} p_{ij,t}=1-{\left(1-{\left(\frac{m_{t}}{M}\right)}^{2}\right)}^{N-2}. \end{array}$$

The value of $p_{ij,t}$ is very sensitive to the link density $d=\frac{m_{t}}{M}$. For large values of *N*, we have that, if $m_{t}∼o\left(N^{\frac{3}{2}}\right),$ then $p_{ij,t}$ remains small, approaching value 1 otherwise. Therefore, the behavior of the model presented in Section 5 may be approximated considering two possible regimes.

1. Regime 1. For large enough $m_{t}∼\Omega \left(N^{\frac{3}{2}}\right),$ the probability of creating or deleting triangles is not negligible and the dynamics of the system are approximately governed by a model following (23) with $u_{1}=\frac{p_{r,n}}{p_{a,n}}$.
2. Regime 2. For small $m_{t}∼o\left(N^{\frac{3}{2}}\right),$ the probability of creating or deleting triangles is small and the dynamics of the system are approximately governed by a model following (23) with $u_{2}=\frac{p_{r,nn}}{p_{a,nn}}$.

Hence, within each regime, the dynamics can be approximated via the baseline model (23).

We now focus on the analysis for the cases where triangle creation and preservation is favored, meaning that both α and β would be larger than 1. Then, $u_{2}=\alpha \cdot \beta \cdot u_{1}>u_{1}$ and several behaviors can be found depending on the concrete selected values for $u_{1},u_{2}$ and α (or β). Precisely, some regime may be transitory, or both may coexist as stationary behaviors depending on the selected initial conditions. In general, regime 1 will be more common since it fits with a wide range of possible values for $u_{1}$; only if $u_{1}$ is very large (note that it would imply a huge $u_{2}$) may we start with a $g_{0}$ satisfying regime 1 condition, but the expected value of stationary distribution $M_{s}$ for such $u_{1}$ may correspond to regime 2, so that the system may end up in such second regime. On the other hand, since regime 2 corresponds to a narrow range of small values of $M_{t}$, a very large value of $u_{2}$ will be required for such regime to show up as stationary; if $u_{2}$ is not large enough, even if we start with a $g_{0}$ satisfying regime 2 condition, the expected value of stationary distribution $M_{s}$ for such $u_{2}$ may lie in the range of values corresponding to regime 1, so that the system may end up in such first regime. Finally, both regimes may coexist with $u_{2}$ large enough and $u_{1}$ small enough so that the respective expected values of stationary distributions $M_{s}$ correspond to each one of the regimes. Note that, if α and β are large enough to favor triangle creation and preservation, $u_{1}$ and $u_{2}$ may differ in some orders of magnitude allowing a natural coexistence of both regimes.

In the next section, different simulations are performed to characterize the time evolution of some basic features (e.g., the expected value for the number of links, the clustering coefficient, the connectivity and the sample degree distribution) for the dynamics models presented above.

## 6. Simulations for the Time Evolution of Features

Numerical simulations have been performed to characterize the time evolution of the number of links, the clustering coefficient and the entropy of the sample degree distribution for the extended model defined by (23) and the structure dependent model presented in Section 5.

### 6.1. Extended Asymmetric Model

**Figure 2:**
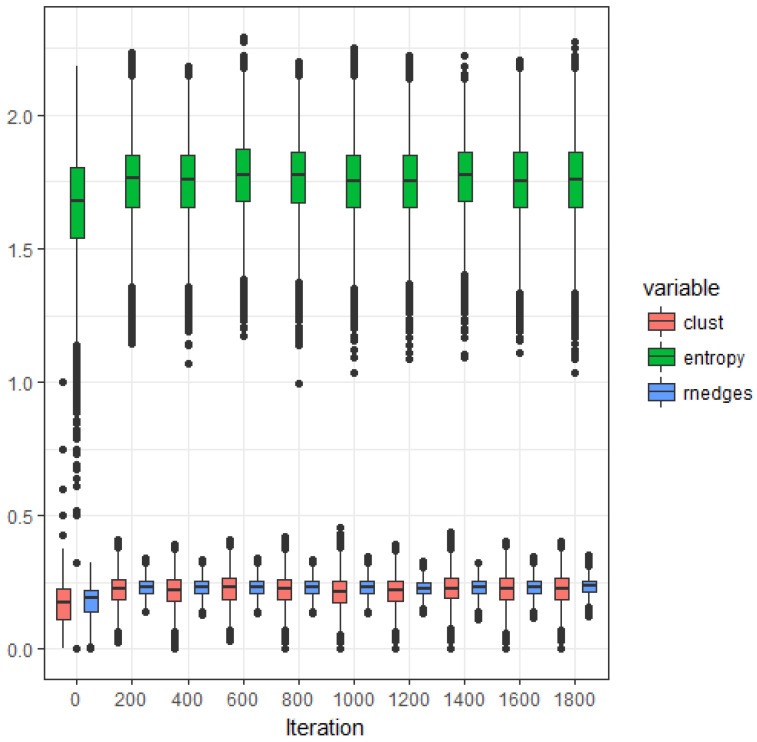
Extended asymmetric model. Estimated expected values of relative number of edges (rnedges), clustering coefficient (clust) and sample degree distribution entropy (entropy), as a function of the iteration number ($p_{a}=0.3$ and $p_{r}=1$).

We begin by characterizing the extended model defined by (23). Figure 2 shows the evolution (starting from the empty graph) of the relative number of edges (i.e., edge density $d=\frac{m_{t}}{M}$), the clustering coefficient and the samples degree distribution entropy of a graph that evolves following the extended model defined by (23) with $p_{a}=0.3$ and $p_{r}=1$. The estimations of relative number of edges and clustering coefficient converge to the same stationary value as the iteration number increases; hence, their ratio converges to one, this value being a reference baseline signature of structure independent models. Note that the variance of the clustering coefficient is significantly larger than the variance corresponding the relative number of edges. The estimated degree distribution presents also a significant variance.

**Figure 3:**
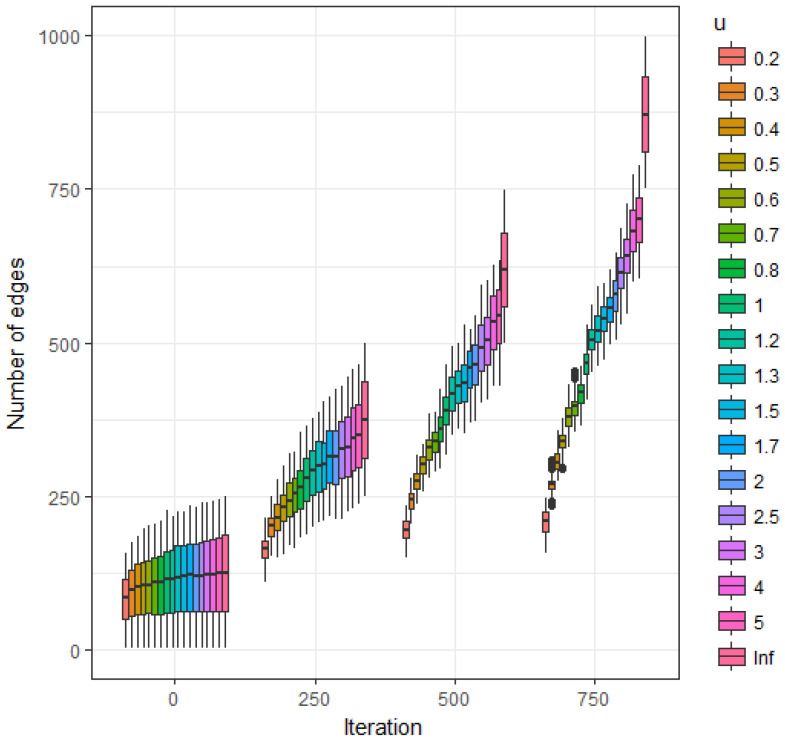
Extended asymmetric model. Estimated expected value of number of edges as a function of *u* at iterations 250, 500, 750 and 1000.

Figure 3 represents the estimated expected value of the number of edges as a function of iteration number (starting from the empty graph) and parameter *u*. Due to the uniform nature of $P(G_{i}|M_{i}),$ the behavior of the clustering coefficient follows a similar behavior; again, this clustering coefficient/link density ratio value close to one is a reference baseline signature for these types of structure independent models.

**Figure 4:**
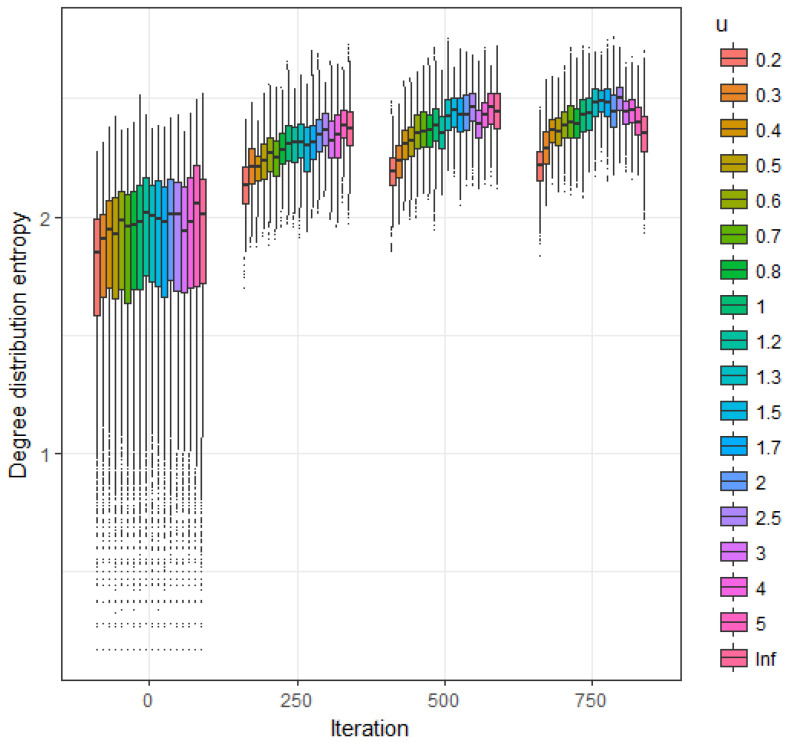
Extended asymmetric model. Estimated expected value of the sample degree distribution entropy as a function of *u* at iterations 250, 500, 750 and 1000.

Figure 4 represents the estimated expected value of the sample degree distribution entropy as a function of iteration number (starting from the empty graph) and parameter *u*. Larger values are obtained for u=1 as also illustrated in Figure 1.

### 6.2. Structure Depending Model

We now illustrate the behavior of some models of the type presented in Section 5. A network with n=100 has been considered, where different parameter values and initial conditions have been tested. In the following, we indicate some cases that illustrate the different behaviors:

1. If $p_{an}=0.5$, $p_{ann}=0.05$, $p_{rn}=0.1$ and $p_{rnn}=1$ (or, equivalently, $u_{1}=20$, $u_{2}=0.2$, α=β=10) the system always converges to a stationary behavior in regime 1, with link density $d=\frac{m_{t}}{M}\approx 0.8$, and clustering coefficient cc≈0.8 (hence the ratio $\frac{cc}{d}$ approaches 1 as it happens in Erdős–Renyi [22] or Gilbert models).
2. If $p_{an}=0.01$, $p_{ann}=0.005$, $p_{rn}=0.5$ and $p_{rnn}=1$ (or, equivalently, $u_{1}=200$, $u_{2}=50$, α=β=2), the system always converges to a stationary behavior in regime 2, with link density $d=\frac{m_{t}}{M}\approx 0.005$, and oscillating clustering coefficient between 0 and 0.2 (hence the ratio $\frac{cc}{d}$ presents also large oscillations).
3. If $p_{an}=0.05$, $p_{ann}=0.005$, $p_{rn}=0.1$ and $p_{rnn}=1$ (or, equivalently, $u_{1}=200$, $u_{2}=2$, α=β=10), the system presents the two above illustrated regimes; and, depending on the initial condition, there is more or less probability to evolve within each one of these regimes:
  (a) For $g_{0}$ following a Gilbert model with p⪆0.0375, the system is more likely to remain in regime 1 with link density $d=\frac{m_{t}}{M}\approx 0.32$, and clustering coefficient cc≈0.32 (hence the ratio $\frac{cc}{d}$ approaches 1 as it happens in ER or Gilbert models).
  (b) For $g_{0}$ following a Gilbert model with p⪅0.0375, the system is more likely to steadily remain (at least for a long time beyond the number of performed iterations) in regime 2 with link density $d=\frac{m_{t}}{M}\approx 0.008$, and clustering coefficient cc≈0.3. Hence, the ratio $\frac{cc}{d}$ evolves around 40; this large clustering coefficient in proportion to link density illustrates a very common feature of many real networks.

**Figure 5:**
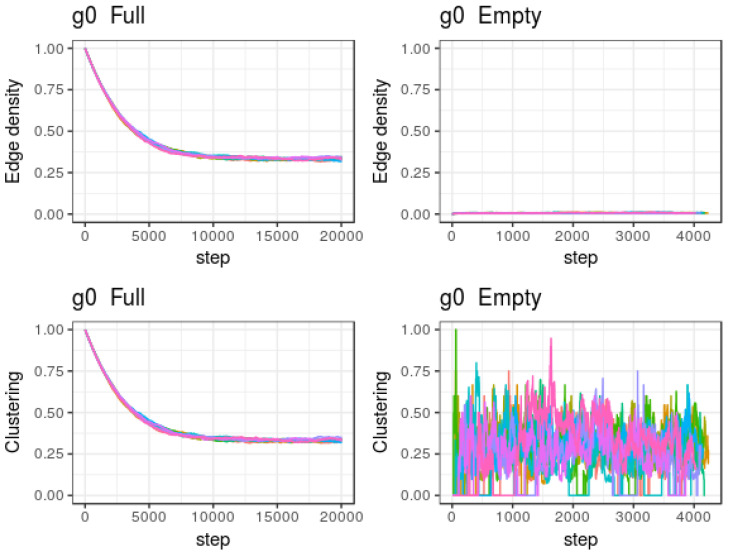
Structure depending model. Estimated expected value of number of edges and clustering coefficient as a function of number of steps, starting from complete and empty graphs, respectively. Different line colors correspond to different instantiations of the model.

**Figure 6:**
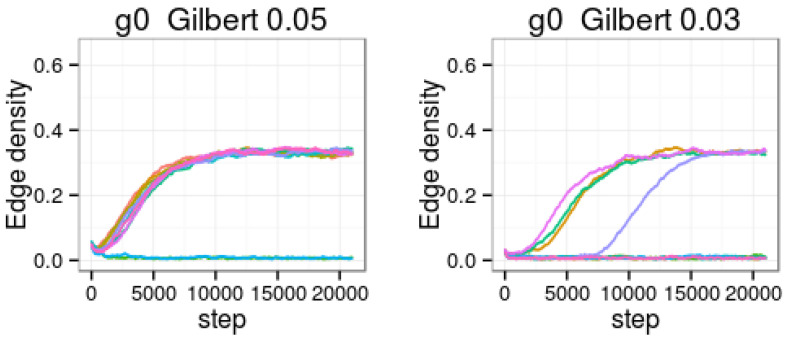

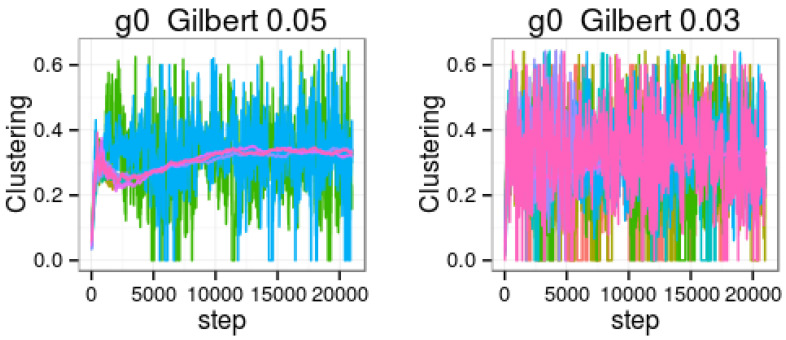
Structure depending model. Estimated expected value of number of edges and clustering coefficient as a function of the number of steps, starting from a sample of the Gilbert model with p=0.05 and p=0.03, respectively. Different line colors correspond to different instantiations of the model.

The two regime case is illustrated in Figure 5 and Figure 6, where the estimated expected value of the density of edges and the clustering coefficient as a function of the iteration number are presented for different initial condition graphs $g_{0}$. Figure 5 illustrates the behavior when $g_{0}$ is either the complete or the empty graph. All simulations starting from the complete graph led to regime 1, whereas all simulations starting from the empty graph led to regime 2.

Figure 6 illustrates the behavior for two cases when $g_{0}$ is obtained as a sample of the Gilbert model with p=0.05 and p=0.03, respectively. A majority of the simulations starting from a Gilbert p=0.05 graph led to regime 1, whereas a majority of the simulations starting from a Gilbert p=0.03 graph led to regime 2. These simulations illustrate approximately the size of the stochastic domains corresponding to each regime.

## 7. Conclusions

Several basic models for dynamic networks, based only on edge density, have been initially proposed and analyzed in terms of the time evolution of the number of links, clustering coefficient, connectivity and entropy of the sample degree distribution; the evolution of these features helps to characterize the proposed models and provides a reference baseline signature to assess more complex behaviors. The proposed model involving network structure presents a more sophisticated behavior and, for some regime, it leads to the generation of a high clustering coefficient/link density ratio when compared with the reference baseline values. This result is promising for the design of network models with tunable clustering coefficient with the aim to replicate some real networks characteristics.

The proposed framework will serve to assess, in a systematic manner, the properties of existing models as well as future more complex models for time evolving networks.
